# Prediction of psychiatric comorbidity on premature death in a cohort of patients with substance use disorders: a 42-year follow-up

**DOI:** 10.1186/s12888-019-2098-3

**Published:** 2019-05-15

**Authors:** Mats Fridell, Martin Bäckström, Morten Hesse, Peter Krantz, Sean Perrin, Anna Nyhlén

**Affiliations:** 1 Department of Psychology, Lund University, SE-22100 Lund, Sweden; 2 Centre for Alcohol and Drug Research, University of Aarhus, Bartholins Allé, 8000 Århus C, Denmark; 3 Department of Forensic Medicine, Lund University Hospital, SE-22185 Lund, Sweden; 4 Department of Psychiatry, Malmo University Hospital, SE-20502, Malmo, Sweden

**Keywords:** Cohort study, Premature death, 42-year follow-up, Drug abuse, Psychiatric comorbidity, Personality disorder, Somatic comorbidity

## Abstract

**Background:**

We need to better understand how the use of different substances and psychiatric comorbidity influence premature death generally and cause-specific death by overdose, intoxication and somatic disorders in people with substance use disorders.

**Method:**

A cohort of 1405 patients consecutively admitted to a Swedish detoxification unit for substance use disorders in 1970–1995 was followed-up for 42 years. Substances were identified by toxicological analyses. Mortality figures were obtained from a national registry. Causes of death were diagnosed by forensic autopsy in 594 patients deceased by 2012. Predictions were calculated by competing risks analysis.

**Results:**

Forty-two per cent of the cohort died during follow-up; more men than women (46.3% vs 30.4%). The standardised mortality ratio (SMR) was calculated as the ratio of observed deaths in males and females in specific age groups in the cohort versus expected deaths in corresponding groups in the general population. SMR was 5.68 for men (CI 95%; 5.04–6.11) and 4.98 (CI 95%; 4.08–5.88) for women. The crude mortality rate (number of deaths divided by number of person observation years) was 2.28% for men and 1.87% for women.

Opiates predicted increased risk of premature death while amphetamine and cannabis predicted lower risk. Comorbid psychiatric disorders were identified in 378 cases and personality disorders in 763 cases. Primary psychoses or mood/depression and anxiety disorders predicted a higher risk of premature mortality.

Death by overdose was predicted by male gender, younger age at admission to substance treatment, opiate use, and comorbid depression and anxiety syndromes. Cannabis and amphetamine use predicted a lower risk of overdose. Death by intoxication was predicted by male gender, use of sedatives/hypnotics or alcohol/mixed substances, primary psychoses and depression/anxiety syndromes. Premature death by somatic disorder was predicted by male gender and alcohol/mixed abuse.

**Conclusion:**

Psychiatric comorbid disorders were important risk factors for premature drug-related death. Early identification of these factors may be life-saving in the treatment of patients with substance use disorders.

## Background

Persons with a substance use disorder (SUD) have an increased risk of dying prematurely compared to age- and gender-matched individuals in the general population. Premature mortality is defined as death occurring before the average age of death within a given population. A study in nine European countries reported an overall mortality ratio of SUD patients abusing illicit drugs that was 10–20 times the level in the general population of the same age and gender [1]. The annual mortality rate was 1–2% and the crude mortality rate per 1000 person years follow-up was 14.2, varying from one country to another from 3.5 to 22.7 [1]. Premature death in SUD patients is associated with intravenous administration of heroin, stimulants like amphetamine, methamphetamine, cocaine or other drugs [2–6]. Types of drugs, young age and poly-drug abuse are known risk factors for increased premature death in substance abusers [1, 6, 7]. There are, however, few studies analysing how different substances and comorbid psychiatric disorders can predict premature death.

In a previous longitudinal study of SUD patients treated in the 1970s, we reported premature mortality for types of SUD and comorbid psychiatric disorders applying ICD-8 and ICD-9 diagnoses [7]. In the present larger 42-year follow-up study, data for the complete cohort of 1405 patients admitted to the treatment unit up to July 1995 was available and diagnoses were updated to ICD-10 for causes of death, substance use and psychiatric disorders [8]. At follow-up, 594 patients were deceased. The comorbid psychiatric disorders were grouped into four different clusters according to nosology, and personality disorder diagnoses according to DSM-III-R and DSM- IV were included [9].

### Drug-related death

#### Illicit opiates and opioids

Opiates were introduced in the major cities in Sweden in the early 1970s, mostly compounds like morphine base. Heroin was introduced on the Swedish street market in 1974. Across Europe, most premature deaths among substance abusing patients are drug-related, with overdose as the major cause of death among heroin users [1]. Some 30–40% of all deaths in opiate users are caused by overdose [5], corresponding to 0.7% death by overdose in epidemiologic studies [6, 10]. Overdose is defined as an instant death following intake of an illicit drug [11] and opiates or opioids contribute more than any other substance to drug-related and overdose death [1]. Overdose may occur also in abuse of substances other than opiates, but to a lesser degree [6]. Meta-analyses estimated the mortality among regular users of illicit opiates to 13 times the norm for their age-peers [5, 12]. The annual mortality rate in clinical cohorts of opiate-dependent patients ranges from 1.2 to 2.2% [1, 13, 14].

The use of illicit substances in Sweden has been quite stable during the 1980s and 90s. A registry update on all obtainable forensic autopsy reports in Sweden, however, estimated a 33% increase of drug-related deaths from 2008 to 2014 [15]. This increase persisted after controlling for changes in methods assessing toxicological data at autopsy. Deaths were mainly caused by opioids like methadone, buprenorphine, fentanyl and oxycodone. Legally prescribed opioids were present at autopsy in about 70–75% of the drug-related deaths, while illicit drugs were less frequent, consistent with findings on drug-related deaths from England and the USA [1, 16]. Opiate use has dominated the drug scene in most Western countries since the 1970s [17–19].

The substances in the present study were the same as those in other clinical studies in Sweden at the same time. An average rate of premature death of 38% was reported in a Swedish cohort study of 1640 drug users treated in a detoxification unit between 1985 and 2007 [14].

**Amphetamines and other stimulants** were the most common substances on the illegal market in Sweden before 1974 and comprised the second largest group of illicit drugs until very recently [7, 14]; they are still highly prevalent in prisons and compulsive treatment units (LVM) [19].

Amphetamines cause drug-related death less often than opiates/opioids [13, 20]. An English database study reported data on substance abuse deaths from central stimulants between 2001 and 2007. Rates of death by amphetamine/methamphetamine and ecstasy were 2.09 and 1.75 per 100,000 users, respectively [20]. In a hospital cohort study of methamphetamine users in Thailand admitted for substance-induced psychoses (SIP), 8.2% died within five years after first admission [21]. In the five-year follow-up of 40% of the patients with an initial substance induced psychosis (SIP), one third had developed a schizophrenia while the other patients had no readmission for psychiatric disorder or psychotic episodes during the five years [21].

**Cannabis** use was associated with an increased risk of premature mortality [4, 22] and increased risk of schizophrenia in earlier Swedish follow-up studies [23]. In a recent 42-year follow-up study of 50,373 Swedish military conscripts, the mortality risk was higher in heavy cannabis users compared to those with no use (HR = 1.4, CI 95%; 1.1–1.8); however, in contrast to earlier findings, there was no interaction between cannabis use or psychotic disorders and mortality compared to a control group [24]. In a Danish cohort of 3114 patients seeking treatment for cannabis use, the incidence of psychiatric disorders was 40.7% compared to 5.2% in age- and gender-matched controls from the general population [4]. Fifteen percent had been treated for a comorbid psychiatric disorder [4]. In a subsequent follow-up study of 6445 cannabis-using patients four years after treatment termination, a low mortality rate of 0.2% was identified with accidents as the most common cause of death (SMR = 8.2; CI 95%; 6.3–10.5) [25].

### Comorbid psychiatric disorders

Psychiatric comorbidity, defined as the co-existence of two or more psychiatric disorders, one of which is substance use disorder, may have a profound impact on outcome and mortality. It is well-known that concomitant substance use in patients with primary psychiatric disorders (i.e. ‘dual diagnosis patients’) has a negative impact on course and outcome. However, dual-diagnosis patients are mostly recruited from psychiatric care populations with most of the patients suffering from a primary psychosis [26, 27].

Substance abusing patients without a primary psychotic disorder present a different clinical picture to those of dual diagnosis patients. In clinical cohorts of substance abusers, as well as in epidemiological studies, about two thirds have at least one comorbid psychiatric disorder [1]. The clinical picture among drug abusers, however, is one with comorbid personality disorders, depression and anxiety syndromes and a smaller percentage of primary psychosis or substance induced psychosis. Psychiatric comorbidity is fairly similar across the different substance groups of illicit substances [17, 18, 28].

The prevalence of psychoses in people with drug-use disorders has been estimated to be below 20% [21, 23–25, 28–30], with psychoses more common in users of cocaine, amphetamine, methamphetamine, cannabis, inhalation abuse and hallucinogenic drugs than in opiate users [18–21, 28].

Differentiating Substance-Induced Psychosis (SIP) from Primary Psychotic Disorders (PPD) is vital when treating patients with substance abuse [31]. The two groups of disorders may share common features at the initial admissions, but those with SIP are characterised by: a) better short- and long-term outcomes; and b) psychotic symptoms abating earlier and permanently when substance use is stopped [31].

Literature reviews report affective/mood disorders and anxiety disorders in 20–60% of substance abusers [13–15]. However, since mood/depression and anxiety disorders are associated with an increased risk of suicide and completed suicide at 5–15% in drug users generally [1, 2, 22] and 3–35% in heroin users specifically [12], these disorders must always be attended to. Another more recent addition to the diagnostic spectrum above is Attention Deficit and Hyperactivity Disorder (ADHD), identified in about 20% of the substance-using patients [29].

### Comorbid personality disorders

Before the publication of DSM-III in 1980, comorbid personality disorders were most often assessed via self-report psychological tests [18] or questionnaires based on the then current ICD-8/ICD-9 criteria [8]. From 1987, structured diagnostic interviews, SCID II were available for assessing DSM-III-R, DSM-IV and subsequent versions [9]. Based on the versions of assessment above, the prevalence of comorbid personality disorders (PDs) in substance users has been estimated at 50–80%, with antisocial and borderline being the most frequent categories [17–19, 28–30, 32–35]. Having at least one PD generally, and anti-social PD in particular, is associated with impulsivity, aggressiveness and high levels of crime of long duration, which complicates the course and remission [19, 26, 28]. Despite the negative impact on treatment compliance in patients with SUD, PDs were seldom included in routine assessments in Swedish substance treatment units [19, 32–35]. The National Epidemiological Survey on Alcohol and Related Conditions (NESARC, *n* = 46,100) in the USA confirmed the findings from clinical cohort studies: higher prevalence of DSM-IV personality disorders in subjects with comorbid drug use disorders (47.7%) and lower prevalence (28.6%) of personality disorders in subjects with alcohol use disorder [36, 37].

### Aims of the study

**–** To compare the rates of premature mortality in a clinical cohort of patients treated for substance dependence with the gender- and age-corrected mortality in the general population in Scania County (Sweden).

– To identify the clinical prevalence of substance dependence disorders (to drug type) and psychiatric comorbidity in a cohort of patients with illicit drug use.

– To evaluate whether type of substance use and psychiatric comorbidity predict premature death in general in a clinical cohort of patients with illicit drug use.

– To evaluate whether type of substance use and psychiatric comorbidity in a clinical cohort of patients with illicit substance use predict cause-specific death by: a) overdose; b) intoxication; and c) somatic disorder.

## Method

### Setting and representativeness of the cohort

The setting was an inpatient detoxification and short-term rehabilitation unit within psychiatry at St Lars University Hospital in Lund (Southern Sweden). It was a typical low-threshold treatment facility in 1970s Sweden, accepting all drug abusers seeking detoxification and treatment for mainly substance use (narcotics), often called ‘street-addicts’. Until the end of the 1990s, hospital treatment with detoxification and short-term rehabilitation was the first-line treatment for drug abuse in Sweden. Almost all SUD patients admitted consecutively to the unit from 1970 to July 1995 were included in the cohort after informed consent.

The catchment area for the unit was the entire Scania County with a population of one million inhabitants in the 1970s. A national case-finding study in 1978 estimated the number of heavy drug (narcotic) abusers at 10,000–14,000 across all of Sweden, with some 2700 using illicit substances daily [38, 39]. Half of the drug using population injected drugs and the majority were between 30 and 39 years of age with 80% being men. The gender, age and primary drug use in the current clinical cohort were comparable to the rates in the case-finding study. A second national case-finding study, conducted in 1998, reported an increase in the number of ‘heavy drug users’ to 28,500 persons in Sweden, with approximately 50% being intravenous users. Of these ‘heavy’ drug users, about 5200 (18.6%) were living in Scania county [40, 41]. The prevalence of heavy drug abuse in Sweden has remained stable over the past fifteen years. In Scania County, the prevalence of heroin use has been continuously high, and was about 30% in 2012 [40–42].

The study was approved by the Ethical Committee of Lund University [LU 22/1983, Dnr 587/2005].

### Participants

A total of 1437 patients were consecutively admitted to the unit for detoxification of narcotic substances from 1970 to 1995, with only 31 patients (2.2%) refusing participation or having missing data. The remaining 1405 patients were followed until death or, at most, 42 years by 31 December 2012 (range 18–42 years, median 30 years). Mortality data and causes of death were obtained from the Swedish Central Bureau of Statistics (SCB) for 594 persons [42].

### Assessment at admission

At admission, substance use and somatic symptoms were assessed as per the routine medical intake carried out by the unit physician. Toxicological analyses of individually supervised urine samples were analysed by thin-layer chromatography or gas chromatography at the laboratory unit of the hospital. Substance data were available in the hospital records for every admission and updated to ICD-10-diagnoses of substance abuse and dependence (F10-F19). In addition, clinical interviews, questionnaires and psychiatric hospital records contained mandatory reporting on length, intensity of substance abuse, psychiatric problems and earlier treatment attempts [39]. Psychiatric diagnoses were conducted by the senior consultant psychiatrist at the unit and psychologists carried out additional psychological assessments. Secondary substance use was not included in the statistical analyses.

### Identifying and coding causes of death

To obtain mortality data, the patients’ national identification numbers were linked to the Swedish Central Person Register (SCB) and the Cause of Death Register (EPC) at the National Department of Health, wherein all deaths are consistently recorded by the Swedish Central Bureau of Statistics (SCB) [42]. The coverage of deaths in Sweden is close to 100% because reporting is mandatory [7].

Forensic autopsies in Sweden always include toxicological analyses when the deceased is a substance abuser with an accidental, unclear or violent death. Toxicological data were studied in detail case-wise to differentiate suspected from confirmed drug-related death. Hospital records and police reports from the actual scene of death supplied additional information.

Autopsy protocols and death certificates were obtained from the forensic clinics in Sweden and Denmark and were coded according to ICD-10 by a senior consultant physician (Anna Nyhlén) and an associate professor of forensic medicine (Peter Krantz). All diagnoses were specified and defined by the coroner. The two researchers classified the first 100 causes of death diagnoses independently of each other and calculated the reliability of drug-related or non-drug related causes of death with a high interrater reliability (*k* = 0.95) [7].

### Defining drug-related and non-drug related death

*Drug-related death* is defined by substance as the underlying cause of death and the primary reason for dying. The definition is adopted from Degenhardt et al. [43] with additional ICD-10 codes for alcohol and for self-inflicted intoxication, or intoxication of unclear intention. Drug use as an underlying cause of death refers to those cases when death is directly associated with drug in autopsy protocols or death certificates. The primary cause of drug-related death is defined by the substances involved and the context.

*Overdose* is defined as acute death, occurring shortly after the intake of an illicit drug, and directly related to the intake [11]. Overdoses are classified by type of drug.

*Intoxication* is an acute death occurring shortly after intake of licit drugs and directly related to the intake. Licit drugs are prescribed sedatives and hypnotics or drugs like Methadone or Buprenorphine in ongoing maintenance treatment. Licit drugs may be mixed with illicit ones.

*Non-drug related death* is classified by the coroner as death caused by somatic disorders or by accident, suicide or other violent means without illicit and/or licit drugs or alcohol being involved.

### Assessing psychiatric comorbidity

Of 1405 patients, 378 (27.5%) had a hospital record that contained a diagnosis of psychiatric disorder. These records were then scrutinised together with laboratory reports by the senior psychiatrist (Per Tätting) and the head of the research project (Mats Fridell). In addition, toxicological data, somatic diagnoses and psychological tests in the hospital records were analysed. In the case of patients with a suspected psychosis, additional evaluations were always carried out by two senior consultants in psychiatry specialised in diagnostics and treatment of psychoses. All ICD-8/9 diagnoses in the hospital records were updated according to ICD-10 [8]. Only psychiatric diagnoses stable over 12 months were used in the current analyses.

ICD-10 diagnoses of psychiatric disorders with 12 months prevalence are displayed in Table 3. These were organised into four clusters depending on type and nosology: primary/chronic psychotic disorders, severe mental illness (**SMI**) [44], substance-induced psychosis (**SIP**) [31], and other mental disorders (**OMD**), mood disorders such as depression, bipolar and anxiety disorders. The cluster with no psychiatric comorbidity diagnosis was labelled no mental illness (**No MI**).

For patients admitted to the unit from 1982 to 1995, clinical interviews were carried out to establish the presence of PDs according to DSM-III, DSM-III-R and DSM-IV criteria. Some patients who had been treated before 1980 were assessed when readmitted and included in the data set. When feasible, data from SCID-II-assessments were applied [9]. The incidence of PDs assessed via clinical interview versus the SCID-II was compared for 138 patients and this revealed a high level of correspondence. The overall PD prevalence in an earlier study was 79% assessed via clinical interview and 80% using the SCID-II [45]. High agreement was found for borderline personality disorder *k* = 0.77, with moderate levels of agreement for any personality disorder *k* = 0.48 and antisocial personality disorder *k* = 0.49 [9].

### Statistics

All data were analysed using the statistical package IBM SPSS 25.0 [46].

Mortality rates in the cohort were compared to mortality in the general population in Scania County for the period 1980–1995, calculating the standardised mortality ratio (SMR) by gender in five-year age groups. SMR was calculated as the ratio of observed deaths in the cohort versus expected deaths in males and females in the general population. All causes of death in the cohort were included until 31 December 2012. Patients who left Sweden (*n* = 30) were censored by date of emigration.

In each five-year age group, the number of patients at risk was calculated as the number who: 1) entered the cohort by the age-interval; 2) had not died or migrated by the specific age-group interval; and 3) had not passed that age-group interval until 31 December 2012. Adjusting for race or ethnicity was not considered necessary as there was little ethnic diversity in Sweden from the 1970s until 2005.

Data are reported in absolute numbers and proportions. Comparisons of proportions at nominal and ranking level used *χ*^*2*^_*−*_ assays. For comparisons of the data reported in intervals or quotas, *t*-tests, one-way analysis of variance or multivariate analyses were applied. Corrections for skewed distributions used Tukey’s honest significant difference test for post-hoc calculations in ANOVA analyses [46].

Data were subjected to a Cox regression analysis with separate analyses for substances and psychiatric comorbidity to study long-term outcome. Competing risks analysis was used to compare the influence of the substances and the psychiatric comorbidity on premature death and type of death. Competing risks analysis enables estimates of the likelihood of an event when other events take place that alter the probability of the event of interest [47]. The predictors of premature causes of death are reported as hazard ratios (HR).

Prediction analyses of substances and psychiatric comorbid diagnoses were first carried out separately for premature death, controlling for age at first treatment episode and sex as co-variates. The final analyses applied predictor analyses on three different causes of death: overdose, intoxication and somatic disorder. Each predictor was analysed with separate simple regressions and was contrasted with all other predictors.

The competing risks analyses were used to estimate the coefficients while testing the alternative causes of death as censored cases. All censored cases were calculated as the number of years at the year for the follow-up. Time is defined by the number of years since the first admission period started to follow up, or time of death for cases in the predicted group. Patients emigrating were censored by date of emigration.

## Results

### Patient characteristics

The final cohort with 1405 patients was followed up to a maximum of 42 years or until date of death or emigration (range 18–42 years). The majority was men (*n* = 988, 70.1%) with a mean age of 26.7 years (SD = 7.5, median = 25 years), and 417 were women (29.9%); mean age 25.9 years (SD = 8.1, median = 24 years) at first admission (*t* = 4.14, *p* = .03). Patients were, on average, treated four times during the study (SD = 5.9). The average treatment time was 69.5 days (SD = 110.6) with a median of 37 days. Nearly one fifth of the patients (19.1%) were treated at least once under the Compulsory Mental Care Act (LSPV/LPT) for an average of 15.5 days (SD = 51.3).

Most patients had a family history of social disruptions in childhood, destructive home conditions, psychiatric disorders and persistent abuse of alcohol and drugs. Boys were introduced to substance use at an average age of 14.6 years (SD = 2.8) and girls at 15.5 years (SD = 3.6); (*t* = 6.82, *p* = .04). The most common onset drugs were cannabis, alcohol or amphetamine. More women than men came from broken homes. There were no significant gender differences for the patients having been admitted to youth psychiatry or presence of psychiatric problems in the family of origin (see Table 1). There was a statistical tendency that men had been sentenced to probation (37% vs 29%) and/or prison (31% vs 18%) more often than women (*χ*^***2***^_***(1)***_ = 2.65, *p* = .10).

**Table 1:** Patient characteristics in the cohort at first admission (*n* = 1405)

	Men [*n* = 988]	%	Women [*n* = 417]	
	n	%	n	%
Age of onset of drug use	14.6 [SD = 2.8]	70.1	15.5 [SD = 3.6]	23.4
Age at first admission	26.7 [SD = 7.5]	70.0	25.8 [SD = 7.9]	30.0
Psych disorders in family	499	50.2	206	50.6
Broken family	283	28.8	141	33.7
Foster care	107	10.8	39	9.6
Adopted	35	3.5	7	1.7
Child/Adolescent psychiatry	158	15.9	57	14.0
Compulsory psychiatric care	243	24.4	103	25.3
Prosecuted/convicted	174	17.5	54	13.3
Prison	305	30.7	75	18.4
Probation	364	36.6	119	29.2
Opiates	340	34.6	136	32.6
Stimulants	313	31.7	148	35.5
Cannabis	189	19.1	35	8.4
Sedatives/Hypnotics/Barbiturates	48	4.9	53	12.7
Hallucinogenes	13	1.3	3	0.7
Alcohol	64	6.5	24	5.8
Polysubstance use	11	1.1	16	3.8

Calculation based on-tests or χ^**2**^_**[1]**_. Substance percentage based on *n* = 1405

At first admission, the average patient had used narcotic substances daily or at least three to four times a week for more than two years, similar to the definition of ‘heavy drug use’ in the two Swedish case-finding studies, which are the reference material [38–40].

In the cohort (*n* = 1, 405), 378 patients (26.9%) had at least one comorbid psychiatric disorder. Psychiatric diagnoses could not be assessed in 274 patients (19.5%) due to short stay or only one admission. Viewed as random dropouts, a prevalence estimate based on 1131 patients who could be assessed, the total adjusted prevalence of any comorbid psychiatric disorder was 33.4%. Calculated from the cohort, 763 patients of 956 assessed (80%) had at least one personality disorder according to DSM-III-R/IV. Only 24.5% of the opiate dependent patients were ever treated in methadone maintenance treatment (MMT) due to the very strict regulations in Sweden before 1990. Half of the patients in MMT were deceased by 2012.

### Mortality and causes of death

The premature mortality in the cohort was almost six times the level in the general population matched for age and gender. SMR for men was 5.68 (CI 95%; 5.04–6.11) and for women 4.98 (CI 95%; 4.08–5.88). Half of the men and one third of the women were deceased by 2012. Crude mortality rate was 2.28% for men (based on 457 deaths and 20,044 observation years) and 1.87% for women (based on 137 deaths and 7326 observation years).

By 31 December 2012, 594 patients were deceased while 811 were alive. Autopsy protocols were obtained for 438 cases (74%) and another 79 death certificates (14%) were issued during hospital admission, with medical records and laboratory reports available for classification. Death certificates from the National Department of Health were obtained for 578 cases (97%). Information on causes of death was missing for 16 patients (3%), who either died outside Sweden with no autopsy, or where diagnosis was not possible due to decomposition.

The primary substance diagnosis identifies the dominating substance use for which treatment was provided. The primary substances used at first admission were opiates (33.8%), amphetamine/stimulants (32.6%), cannabis (15.9%) and sedatives/hypnotics (8.9%). Other substances like hallucinogens or solvents were used in only 2.2%. At admission, 6.1% had a primary diagnosis of alcohol dependence. Cannabis use was more common in men than in women (19.4% vs 8.4%; *χ*^*2*^_*(1)*_ *=* 21.22, *p* < .001). No sex differences were found for use of sedatives and hypnotics.

Intoxication was the most frequent diagnosis of all causes of death (27.4%) followed by overdose (24%). In opiate users specifically, overdose was the cause in 61.4% followed by intoxication (32.5%).

### Substances predicting premature death

The hazard ratio for premature mortality was estimated somewhat larger for every year older the subject was at first treatment (*p* < .001). The HR for men was much larger than for women (p < .001) (see Table 2). The two variables were used as covariates in the prediction of premature death.

**Table 2:** Prediction of primary substance dependence at admission on premature death^a^

	Wald	p	HR	95% CI for HR	
				Lower	Upper
Age	69.99	0.001	1.04	1.030	1.05
Gender	20.04	0.001	1.55	1.28	1.87
Cannabis	17.004	0.001	0.57	0.43	0.74
Opiates	11.359	0.001	1.34	1.13	1.58
Central stimulants	4.893	0.027	0.82	0.69	0.98
Sedatives/Hypnotics/Barbiturates	1.174	0.279	1.19	0.87	1.63
Hallucinogens	0.877	0.349	0.66	0.27	1.58
Alcohol	5.14	0.023	1.41	1.05	1.90
Mixed drugs	6.245	0.012	1.87	1.15	3.06

^a^Each predictor was analyzed with separate simple regressions and was contrasted with all other predictors

The substances predicting a higher risk of premature death were opiates (HR = 1.34, p < .001), alcohol (HR = 1.41, *p* = .023), and mixed drugs (HR = 1.87, *p* = .012). Amphetamine (HR = 0.82, *p* = .03) and cannabis use (HR = 0.57, *p* < .001) predicted a lower risk of premature death.

### Psychiatric comorbidity as predictor of premature death

The psychiatric diagnoses are displayed in Table 3, and the four diagnostic clusters delineated as above have been used for predicting the impact of psychiatric comorbidity on premature mortality and different causes of death: a) severe mental illness (**SMI**) [44]; b) substance-induced psychosis (**SIP**) [31]; c) other mental disorders (**OMD**), mood disorders such as depression, bipolar and anxiety disorders. The cluster with no psychiatric comorbidity was labelled: d) no mental illness (**No MI**). The proportions of patients deceased in each cluster were: SMI 53.2%, SIP 36.7%, OMD 46.6%, and No MI 40.6%.

**Table 3:** Comorbid diagnostic diagnoses and clusters ICD-10, *n* = 387

Severe Mental Illness [Chronic psychoses]			Substance induced psychoses		
SMI	Type of disorder	n	SIP	Toxic substance	n
F 20	Schizophrenia	58	F 11.5	Opiates	0
F 22	Chronic delusional	5	F 12.5	Cannabis	11
F 25	Schizoaffective disorder	14	F 15.5	Stimulants	24
F 29	Non-organic psychosis	8	F 16.5	Hallucinogens	2
F 30.2	Manic psychosis	1	F 18.5	Solvents	3
F 31.2, F 31.5	Bipolar psychosis	10	F 19.5	Multiple drugs	29
F 33.3	Recurrent depressive psychosis	14	^a^F23.2, F 23.3, F 23.9		10
	Total	111			79
Other mental disorders [OMD]					
Affective disorders/mood disorders					n
F 30.0–30.1, F31.0–31.1, F31.3–31.4, F31.6–31.9		Bipolar without psychosis			34
F32.0–32.2, F 32.4–32.9		Depression			29
F33.0–33.2, F 33.4–33.9		Recurrent depression			50
F 34.0–34.9		Chronic mood disorder			33
F 39.9		Unspecified mood disorder			1
		Total affective/mood disorders			147
Anxiety disorders					
F 40.0 – F 40.9		Phobias			2
F 41.0 – F 41.9		Anxiety disorders			47
F 42.0 – F 42.9		Compulsive disorders			3
F 43.0 - F 43.2		PTSD, Stress disorders			5
F 44.0 – F 44.9		Dissociative disorders			1
F 50.0 – F 50.9		Eating disorders			2
		Total Anxiety disorders			60
Total [OMD]					207

^a^F 23.2, F 23.3, F 23.9; Acute, transient psychotic symptoms with short duration

Table 4 displays the hazard ratios for the four clusters. Two clusters of comorbid psychiatric disorders predicted a higher risk of premature death: SMI (HR = 1.40, *p* = .015) and OMD (HR = 1.45, *p* < .001). No psychiatric disorder (‘No MI’ cluster) predicted a lower risk of premature death at follow-up (HR = 0.74, *p* < .001). The SIP cluster did not predict premature death.

**Table 4:** Prediction of psychiatric comorbidity on premature death^a^

	Wald	p	HR	95% CI for HR	
				Lower	Upper
No psychiatric disorder (No MI)	10.940	0.001	0.74	0.62	0.89
Substance induced psychosis (SIP)	1.340	0.247	0.80	0.54	1.17
Severe Mental Disorder (SMI)	5.549	0.015	1.40	1.07	1.85
Other mental disorders (OMD)	10.751	0.001	1.45	1.16	1.82
Any personality disorder	1.231	0.267	1.05	0.90	1.10
DSM-IV-cluster II	1.902	0.762	0.99	0.97	1.02

*No MI*; no mental illness/ psychiatric disorder added to substance abuse. *SIP*; substance induced psychosis. *SMI*; severe mental illness with psychosis. *OMD*; other mental disorder without psychotic symptoms. DSM-IV-cluster II: histrionic, narcissistic, borderline and antisocial personality disorders

^a^Each predictor was analyzed with separate simple regressions and was contrasted with all other predictors

Comorbid DSM III-R/IV personality disorders were assessed in 956 cases, of which 20% had no PD. Anti-social PD was the most prevalent category, followed by borderline, histrionic and narcissistic PDs. Among patients assessed, the prevalence of any PD was 80%. PD did not predict premature death (see Table 4).

### Predictors for cause-specific mortality

Tables 5–7 present data on the analyses for three causes of death: overdose, intoxication, and somatic disorder. Gender and age at admission were used as covariates.

**Table 5:** Prediction of overdose death by substance use and comorbid psychiatric disorders^a^

	Wald	p	HR	95% CI for HR	
				Lower	Upper
Gender	10.546	0.001	2.03	1.33	3.12
Age at first admission	1.99	0.003	0.96	0.94	0.99
Cannabis	10.926	0.001	0.35	0.19	0.65
Opiates	45.332	0.001	3.27	2.32	4.62
Central stimulants	7.950	0.005	0.57	0.38	0.84
Sedatives/hypnotics/Barbiturates	1.398	> 0.05	0.54	0.20	1.50
Alcohol mixed with other subst.	2.508	> 0.05	0.48	0.20	1.19
No psychiatric disorder (No MI)	0.949	> 0.05	0.83	0.58	1.24
Substance induced psychosis (SIP)	2.218	> 0.05	0.47	0.17	1.27
Severe Mental Disorder (SMI)	0.112	> 0.05	0.90	0.47	1.78
Other mental disorders (OMD)	7.225	0.007	1.80	1.17	2.77

*No MI*; no mental illness/ psychiatric disorder added to substance abuse. *SIP*; substance induced psychosis. *SMI*; severe mental illness with psychosis. *OMD*; other mental disorder without psychotic symptoms

^a^Each predictor was analyzed with separate simple regressions and was contrasted with all other predictors

**Table 6:** Prediction of death by intoxication by substance use and comorbid psychiatric disorders^a^

	Wald	p	HR	95% CI for HR	
				Lower	Upper
Gender	3.891	0.05	1.44	1.00	2.06
Age at first admission	1.461	> 0.05	0.99	0.97	1.01
Cannabis	3.102	> 0.05	0.65	0.41	1.05
Opiates	0.000	> 0.05	0.99	0.72	1.39
Central stimulants	2.216	> 0.05	0.77	0.55	1.09
Sedatives/hypnotics/Barbiturates	6.036	0.014	1.94	1.11	3.30
Alcohol mixed with other subst.	8.569	0.003	1.97	1.25	3.10
No psychiatric disorder (No MI)	9.846	0.002	0.60	0.43	0.82
Substance induced psychosis (SIP)	0.606	> 0.05	0.44	0.35	1.58
Severe Mental Disorder (SMI)	3.847	0.05	1.62	1.00	2.62
Other mental disorders (OMD)	9.614	0.002	1.85	1.25	1.72

*No MI*; no mental illness/ psychiatric disorder added to substance abuse. *SIP*; substance induced psychosis. *SMI*; severe mental illness with psychosis. *OMD*; other mental disorder without psychotic symptoms

^a^Each predictor was analyzed with separate simple regressions and was contrasted with all other predictors

**Table 7:** Prediction of death from somatic disorders by substance use and comorbid psychiatric disorders^a^

	Wald	p	HR	95% CI for HR	
				Lower	Upper
Gender	3.476	0.05	1.34	0.99	1.83
Age at first admission	0.802	> 0.05	0.99	1.00	1.01
Cannabis	1.826	> 0.05	0.73	0.41	1.15
Opiates	0.030	> 0.05	0.97	0.72	1.32
Central stimulants	0.375	> 0.05	0.91	0.67	1.23
Sedatives/hypnotics/Barbiturates	0.156	> 0.05	0.69	0.68	1.80
Alcohol mixed with other subst.	6.505	0.01	1.71	1.13	2.57
No psychiatric disorder (No MI)	1.497	> 0.05	0.82	0.60	1.13
Substance induced psychosis (SIP)	0.240	> 0.05	1.15	0.65	2.03
Severe Mental Disorder (SMI)	3.147	> 0.05	1.53	0.96	2.43
Other mental disorders *(*OMD*)*	0.015	> 0.05	0.98	0.63	1.51

*No MI*; no mental illness/ psychiatric disorder added to substance abuse. *SIP*; substance induced psychosis. *SMI*; severe mental illness with psychosis. *OMD*; other mental disorder without psychotic symptoms

^a^Each predictor was analyzed with separate simple regressions and was contrasted with all other predictors

The risk of overdose death (see Table 5) was higher in men than in women (HR = 2.03, *p* < .001), in opiate versus users of other substances (HR = 3.27, p < .001), and in patients with a lower rather than a higher age at admission to treatment (HR = 0.96, *p* < .003). A lower risk for overdose death was found in amphetamine users (HR = 0.57, *p* < .005) and in cannabis users (HR = 0.35, *p* < .001).

Psychiatric disorders without psychotic symptoms (OMD) predicted a higher risk of death by overdose (HR = 1.80**,** *p* < .007), while there was no association to overdose death in the other clusters: SIP, SMI, and No MI.

Intoxications were mostly caused by legal and/or prescribed drugs/medications. Table 6 displays the Cox regression for death from intoxication.

A higher risk of death by intoxication was predicted by male gender (HR = 1.44, *p* = .05), use of sedatives/hypnotics/barbiturates (HR = 1.94, *p* = .014), and alcohol/mixed substances (HR =1.97, *p* < .003). The SMI cluster predicted a higher risk of death by intoxication (HR = 1.62, *p* < .05) as did the OMD cluster (HR = 1.85, *p* < .002). Not having a psychiatric disorder (No MI) predicted a lower risk of death by intoxication (HR = 0.60, *p* < .002).

Table 7 presents the variables predicting death from somatic disorders.

There was a higher risk of dying from a somatic disorder in men (43.8%) compared to women (34.4%) (HR = 1.34, p < .05). Alcohol mixed with other substances predicted premature death by somatic disorders (HR = 1.71, *p* = .02).

## Discussion

Almost half of the heavy substance abusers in the cohort died prematurely during the 42-year follow-up. Two thirds had a drug-related death with opiates as the single strongest predictor. Overdose and intoxication caused half of all deaths.

One important aim of this study was to report on premature mortality in a clinical cohort of ‘street addicts’ who entered treatment in the specific hospital unit in Southern Sweden between 1970 and 1995. The unit was the only one in Southern Sweden with a regional uptake for users of narcotics at the time. Most patients with heavy drug abuse problems in the region were treated in the unit at least once and were consecutively included in the cohort by date of admission. The cohort could thus be used for estimating the clinical prevalence of different substances and comorbid psychiatric disorders and to explore whether these factors could predict premature death and cause-specific death.

In the present cohort, two thirds of the patients at admission abused opiates or amphetamines, at admission with the same proportions in males and females. Intravenous administration was the common practice. Cannabis was the main substance in a fifth of the male patients and a tenth of the females. The observed pattern of drug use in this cohort was similar to the rates reported in national surveys of drug use in Sweden [38–40] and comparable to figures in other large detoxification units in Sweden at the same time [14, 28]. It is important to note that this is a cohort of patients who were treated for drug abuse and not alcohol abuse. Alcohol was the primary dependence in only 6% of all cases at admission.

Despite amphetamine being the most abused illicit substance in Sweden until recently and common in this cohort, it was less often fatal [15, 42]. Amphetamine use in the present cohort was associated with a lower risk of premature death than opiates despite intravenous administration being the common practice in both groups of substance users. This is consistent with international studies [6]. The major difference in overdose death is the toxic depressive effects on respiration in the opiates/opioids which is not present in amphetamine overdoses. The intravenous administration per se seems to be less important.

Combining opiates and sedatives increased in drug user fatalities over the time span [15] and more than one substance was detected for two thirds of the patients at autopsy. Alcohol and/or sedatives mixed with opiates were detected in 24% of the drug-related deaths even though alcohol was seldom defined as the underlying cause of death by the coroner. The combination of opiates and benzodiazepines or alcohol was present in many overdoses and intoxications in this cohort. This accords with other studies [7, 13, 48, 49] reporting that, in the absence of a lethal dose of opiates or opioids, the individual dies from a fatal respiratory arrest caused by the combination of sedatives or alcohol with opiates [7, 50]. This particular complication must be taken seriously by the treatment providers since the risk of fatal overdose and intoxication is increased also by prescribed drugs [13, 16].

In the analyses of cause-specific mortality, some distinct factors differentiated death by intoxication from overdose or somatic disorders. Overdose was predicted by male gender, opiate use, being young at first admission, and by comorbid mood and anxiety disorders. In contrast, death by intoxication was predicted by licit drugs like alcohol or prescribed drugs such as sedatives and hypnotics. Other predictors were male gender, primary psychosis and mood and anxiety syndromes. Licit drugs and prescribed medications are important pharmacological treatment of comorbid psychiatric disorders and caused many intoxications in this cohort. The availability of potent drugs should alert the staff for the need of close surveillance and follow-up in order to avoid premature death by intoxication.

Finally, two thirds of the non-drug related deaths in the entire cohort were caused by somatic disorders, similar to other studies [1, 7]. Substance use always expose the person for risk of contracting severe somatic disorders like liver diseases as hepatitis, heart failure and epilepsy [51]. These findings illuminate the need for diagnostic routines and therapy also for somatic comorbid disorders. Male gender and the mix of alcohol and other drugs were the predictors of death by somatic disorders.

The observed pattern of psychiatric comorbidity in the present study is similar to other studies of illicit substance users [17–19, 28, 34, 35]. The major disorders in this group are personality disorders, depressive and anxiety syndromes, and a small proportion of psychotic disorders. The present study was conducted within a specialised psychiatric unit where the risk that a psychosis or other severe psychiatric disorder should pass unnoticed was minimal. Consequently, the rate of psychiatric disorders seems accurate, realistic and robust [21, 25, 28, 30].

Another important finding, if not surprising, was that patients with primary psychoses had the highest rates of premature death (53.2%), while patients with substance induced psychoses had the lowest (36.7%), lower than the patients without comorbidity.

In reviews of clinical SUD samples, mood and anxiety-disorders varies from 20 to 60% [14, 17, 18, 25, 33]. The wide range of variation indicates that the prevalence reported in many studies may be influenced by the differences in severity ratings and diagnostic routines between the studies. With a 12-months prevalence applied like in the present cohort study, 14.7% had comorbid mood and anxiety disorders. These disorders predicted death by overdose as well as by intoxication. The present findings are clinically relevant, since depression and anxiety are often causing long-term suffering and increased risk of suicide [1, 2, 12, 22]. Evidence-based psychosocial treatments should therefore always be considered in addition to pharmacological interventions.

PDs has been the most common disorder in many studies of drug-abusing patients [18, 19, 28, 34–36]. This high proportion of PDs was reported in reviews long before the introduction of DSM-III and subsequent versions [17, 18]. Of 956 patients assessed for PD, 763 (80%) had at least one PD. This proportion in the cohort was present even though PDs diagnosed by ICD-8 and ICD-9 were not included. Registry data in the Swedish national registry of psychiatric disorders reported that less than 20% of the drug abusing patients had a PD compared to 80% in the present cohort. This indicates a large under-reporting of PDs [42], implying that studies based on registry data exclusively may be less representative for estimating prevalence of PDs in drug abusers.

The presence of comorbid PDs in a drug context is nevertheless often complicated by the patient’s impulsivity, aggressiveness and low compliance [28–30, 32, 34, 35]. Consequently, the large proportion of PDs among substance abusers implies the need for a treatment milieu that provides a high level of structure [19, 33–35].

The high presence of a criminal lifestyle associated with illicit drug use and anti-social PD is a complicating, if not a lethal, factor in substance abusing populations [17–19, 32, 34, 35]. Both men and women in the present cohort had served many prison sentences and probations already by the time of first admission. Anti-social PD was a common denominator in previous reviews [17, 34], even if it did not affect premature mortality. One explanation is that personality disorders sometimes serve as a protective factor when periods of incarceration and probation may increase the number and length of drug-free periods in this group, thereby decreasing the risk of exposure to persistent drug use and premature death [18, 37].

### Strengths and limitations

This study benefits from a well-defined, almost complete clinical cohort of substance abusers admitted consecutively over several decades, assessed and treated within the same research-oriented dependence unit. The study reflects the most common illicit and licit substances in a ‘street addicts’ population and related comorbid psychiatric disorders. The assessment of psychiatric disorders and personality disorders and their relation to premature death also brings significant bits of information to the literature.

A unique feature of this Swedish cohort study relative to cohorts recruited in other countries is that only a few of the opiate-dependent patients ever received MMT, and MMT thus did not influence the mortality in the present cohort. This is interesting, given that many longitudinal studies of opiate users are unable to disentangle the risk of overall mortality from the risk of early mortality (primarily from overdose) during the induction and withdrawal phases of treatment with methadone, buprenorphine or other medications.

The impact of psychiatric comorbidity on mortality patterns in substance abusing patients has, to our knowledge, not previously been systematically reported. The findings on personality disorders is another important, if negative, addition to the discourse, illustrating that comorbid personality disorders, while causing problems in treatment and management in general, did not influence premature death. Autopsy data on causes of death with high validity, finally provides – together with registry data on premature mortality over four decades – findings that cannot be considered as coincidental. In contrast to studies based exclusively on registry data the use of autopsy protocols in the present study increase the validity. In a previous study, using registry data alone, drug-related death was underestimated by 37% and suicide by 85% compared to the autopsy protocols for the same individuals [7]. This was avoided in the present study by using data from forensic autopsies.

Despite the validity and completeness of data, there may nevertheless be a risk of underestimating psychiatric disorders collected in a naturalistic setting like the present one. It was not possible to diagnose comorbid psychiatric disorders in 20% of the whole cohort for practical reasons – few admissions and/or short stays. We regard these dropouts as randomly distributed across the cohort.

Care must be taken when discussing causal relationships in a naturalistic cohort design like the present one. However, the combination of long-term follow-up, prediction statistics, diagnostics and comparisons with other similar studies gives a reasonably robust support to our conclusions. The findings seem stable and plausible.

Another limitation in this study is that the substance categories in the prediction (Cox) models used are not independent. They are all compared separately with all other predictors. In this population, there is no obvious comparison group without substance abuse. Therefore, it was not possible to create a control group. The interpretation of the hazard ratios should be to what extent a group is at hazard to premature death compared to all other substance groups. An alternative strategy would have been to select one group as a contrast to the others. However, this should have led to more difficulties when interpreting the hazard ratios.

## Conclusions

Almost half of the substance abusers of illicit drugs died prematurely during the 42-year follow-up. Two thirds died a drug-related death with opiates as the single strongest predictor. Overdose death was predicted by opiate use, male gender and comorbid mood disorders while patients diagnosed with primary psychoses abusing alcohol/sedative-hypnotics died from intoxication. Premature death from somatic disorders was predicted by male gender and alcohol/mixed abuse.

One third of the individuals of the cohort had one or more comorbid psychiatric disorders which predicted premature death. Personality disorders and substance induced psychoses, however, did not. Patients with no psychiatric comorbidity survived to a greater extent.

The findings are most relevant for patients in treatment for persistent substance use disorders, often combined with a criminal lifestyle. The present cohort is representative of Swedish adult abusers of narcotic substances admitted to substance use treatment. The results indicate which comorbidities should be attended to in treatment in order to decrease the risk of premature death.
